# Soy Protein Isolate Supplementation Favorably Regulates the Fermentation Characteristics of *Debaryomyces hansenii* and Flavor Profile in a Sausage Model

**DOI:** 10.3390/foods14111840

**Published:** 2025-05-22

**Authors:** Wenwen Duan, Qiujin Zhu, Jing Wan

**Affiliations:** 1 School of Liquor and Food Engineering, Guizhou University, Guiyang 550025, China; dwwyfd@163.com; 2 Key Laboratory of Agricultural and Animal Products Storage and Processing, Guizhou University, Guiyang 550025, China; ls.qjzhu@gzu.edu.cn

**Keywords:** *Debaryomyces hansenii*, fermented sausage, carbon and nitrogen sources, protease activity, free amino acids, free fatty acids, volatile flavor compounds, electronic tongue, fermentation optimization

## Abstract

The metabolic activity of fermentative microorganisms plays a critical role in determining the flavor profile of fermented meat products. Modulating carbon and nitrogen sources represents a promising strategy for enhancing product quality. In this study, *Debaryomyces hansenii* strains isolated from dry-cured ham were assessed in a sterile sausage model to evaluate the effects of different carbon sources (sucrose, corn starch) and nitrogen sources (leucine, soy protein isolate) on colony growth, enzyme activity, and physicochemical properties. These nutritional factors significantly affected the fermentation performance of *D. hansenii*. Corn starch and soy protein isolate increased colony count by 14.94% and 90%, respectively, and enhanced protease activity by 2-fold and 4.5-fold. Both treatments maintained high lipase activity (>50 U/g). Both supplements improved the water-holding capacity and decreased the water activity. Carbon sources reduced the medium pH, whereas nitrogen sources contributed to the maintenance of pH stability. A further analysis indicated that corn starch promoted the accumulation of aldehydes and ketones, which intensified the sourness and suppressed the saltiness. In contrast, soy protein isolate increased the abundance of free amino acids associated with umami and sweetness, and stimulated the formation of esters, ketones, and pyrazines, thereby enhancing flavor richness and umami intensity. Both ingredients also reduced saturated fatty acid levels and increased the unsaturated to saturated fatty acid ratio. Soy protein isolate exhibited a more pronounced effect on *D. hansenii* fermentation. This study provides a technical reference for enhancing the flavor characteristics of fermented meat products via the adjustment of carbon and nitrogen sources to regulate *D. hansenii* fermentation.

## 1. Introduction

Fermented sausage is a representative of traditional fermented meat products, with a unique flavor primarily resulting from the metabolic activities of microorganisms. In traditional natural fermentation, the random colonization of environmental microorganisms led to variability in product quality across batches. Modern fermentation technology ensures controlled flavor and stable quality through targeted inoculation with fermentation strains such as lactic acid bacteria, staphylococci, and yeasts [1]. Among these strains, *Debaryomyces hansenii* (*D. hansenii*) is a promising yeast strain for enhancing the flavor of fermented meat products. The inoculation of *D. hansenii* into low-sodium fermented sausages made from boar meat can effectively mask boar taint by generating volatile compounds with fruity and cheesy aromas [2]. In dry-cured pork belly, inoculation with *D. hansenii* has also been shown to promote muscle protein degradation, enhance amino acid accumulation, and facilitate the formation of aldehydes, esters, alcohols, ketones, and organic acids [3]. These flavor-enhancing effects are generally attributed to the yeast’s growth performance, enzymatic activities, and metabolite production [4]. Accordingly, enhancing the fermentation characteristics of *D. hansenii* is considered a promising strategy for improving the sensory quality of fermented meat products.

Modern food biotechnology has improved microbial fermentation performance through strain enhancement approaches such as directed evolution, gene editing, and adaptive evolution targeting tolerance to high osmotic pressure or extreme pH conditions [5]. However, long development cycles and high validation costs often limit these strategies. In contrast, supplementing fermentation substrates with conventional carbon and nitrogen sources offers a practical alternative with lower technical barriers and shorter implementation timelines [6]. Carbon and nitrogen sources not only serve as essential sources of energy and nutrients for microorganisms but also regulate their metabolic pathways. Studies have shown that the microbial utilization of these nutrients is controlled by catabolite carbon repression (CCR) and nitrogen catabolite repression (NCR) mechanisms [7]. The CCR mechanism enables microorganisms to preferentially utilize high-efficiency carbon sources (e.g., glucose) while repressing the expression of genes involved in the metabolism of secondary carbon sources (e.g., lactose and maltose), thereby improving energy utilization efficiency. The NCR mechanism regulates nitrogen utilization by repressing the metabolic pathways of secondary nitrogen sources (e.g., proline and urea) when preferred nitrogen sources (e.g., ammonium ions and glutamine) are abundant, and by activating alternative nitrogen utilization pathways under nitrogen-limited conditions [8].

Furthermore, the properties of carbon and nitrogen sources can directly affect the physicochemical characteristics and sensory properties of fermented meat products. For instance, corn starch, owing to its strong water-binding capacity, can improve moisture retention and enhance the structural integrity of the final product [9]. Soy protein isolate, characterized by its high protein content and low fat composition, is widely used to improve water retention, reduce fat content, and facilitate the development of plant-based meat products [10]. In addition, carbon and nitrogen sources can indirectly influence product quality by modulating microbial metabolic pathways. For example, sucrose can increase oxidative stress in staphylococci, thereby promoting the formation of nitrosyl myoglobin, which enhances the color of dry-cured sausages [11]. Branched-chain amino acids (leucine, isoleucine, and valine) can be metabolized by microorganisms into aldehydes, alcohols, and organic acids, contributing to the malt-like and nutty aromas of fermented meat products [12]. The type of carbon and nitrogen sources can affect the accumulation of flavor precursors and final flavor compounds by modulating microbial metabolic pathways. For example, xylose has been shown to promote acetoin production by *Leuconostoc mesenteroides* while inhibiting diacetyl accumulation, whereas the metabolism of fructose and sucrose is closely associated with mannitol production by *L. mesenteroides* [13]. The addition of soy protein isolate and wheat gluten as nitrogen sources has been reported to increase ketone levels in fermented fish paste, thereby enhancing cheese-like flavor characteristics [14].

Current research on *D. hansenii* has primarily focused on its role in enhancing the flavor of fermented meat products, whereas studies investigating the effects of carbon and nitrogen sources on its fermentation performance remain limited. In this study, four carbon and nitrogen sources commonly used in fermented meat products were selected: sucrose and corn starch as simple and complex carbon sources, and leucine and soy protein isolate as small- and large-molecule nitrogen sources. A sterile sausage model was employed to evaluate their effects on the growth, enzymatic activity, and physicochemical properties of a local *D. hansenii* strain. Corn starch and soy protein isolate, which demonstrated the best overall performance, were subsequently selected for the further analysis of flavor compound accumulation. The objective of this research is to explore how carbon and nitrogen sources impact flavor formation by *D. hansenii* during fermented sausage production and to offer practical strategies for optimizing fermentation through formulation design and microbial nutrient regulation.

## 2. Materials and Methods

### 2.1. Preparation of Fermentation Agent

The *D. hansenii* strain used in this study was previously isolated from local dry-cured Panxian ham by the research group and was identified via 26S rDNA sequencing (GenBank accession number: PV249420). The strain was inoculated into Yeast Extract Peptone Dextrose (YPD) broth and incubated at 180 rpm for 48 h. The culture was then centrifuged at 5000× *g* for 5 min at 4 °C, and the harvested cells were washed twice with sterile 0.9% saline. The cell density was adjusted to 1 × 10^8^ cells/mL using a hemocytometer and stored at 4 °C until use.

### 2.2. Preparation of Sterile Sausage Medium

The raw materials used in this study were fresh pork back fat and hind leg meat, obtained from Guizhou Tainong Xingwang Food Co., Ltd. (Guiyang, China). The back fat was sterilized by autoclaving at 121 °C for 15 min. The hind leg meat was sterilized by immersion in 75% ethanol for 10 min, followed by two rinses with sterile water and then exposure to ultraviolet light for 10 min to minimize protein denaturation [15]. Following sterilization, the back fat and hind leg meat were minced and blended at a 3:7 (*w*/*w*) ratio. A sterile solution of 3.0% NaCl, 0.015% NaNO_2_, and 0.05% ascorbic acid (sterilized by 0.22 μm membrane filtration) was added to prepare the sausage medium. A control group (CK) was prepared without additional carbon or nitrogen sources. Sucrose (CS) and corn starch (CC) served as the carbon source treatments, while leucine (NL) and soy protein isolate (NP) were used as the nitrogen source treatments. The carbon and nitrogen sources were added at 5% (*w*/*w*) for all treatment groups, based on the studies by Prestes et al. and Huang et al., with slight modifications [16,17]. Each group was inoculated with 2% *D. hansenii* cell suspension and fermented at 28 ± 0.5 °C with 85% relative humidity for 5 days. After fermentation, the samples were designated as FCK, FCS, FCC, FNL, and FNP for subsequent analyses.

### 2.3. Colony Count

Samples (5 g) from each group were homogenized with 45 mL of 0.9% sterile saline using a YM-08X stomacher homogenizer (Shanghai Yuming Instrument Co., Ltd., Shanghai, China) for 2 min. After appropriate serial dilutions, 100 μL of each dilution was plated onto YPD agar plates. The plates were incubated at 28 ± 0.5 °C for 5 days, and the number of colony-forming units per gram of sample (CFU/g) was determined.

### 2.4. Determination of Enzyme Activity

Protease activity was measured according to the method described by Ding et al. [14]. Samples (5 g) were mixed with 25 mL of phosphate buffer (10 mM, pH 6.8) and homogenized in an ice-water bath for 60 s at 5000 rpm using an XHF-D homogenizer (Ningbo Xinzhisheng Biotechnology Co., Ltd., Ningbo, China). The homogenate was centrifuged at 10,000× *g* for 20 min at 4 °C, and the supernatant was collected as the crude enzyme extract. Subsequently, 1 mL of the crude enzyme was mixed with 1 mL of 2% (*w*/*v*) casein solution and incubated at 40 °C for 10 min. The reaction was terminated by adding 2 mL of 0.4 mol/L trichloroacetic acid. After centrifuging at 10,000× *g* for 20 min at 4 °C, 1 mL of the resulting supernatant was mixed with 5 mL of 0.4 mol/L Na_2_CO_3_ and 1 mL of Folin reagent. After a 20 min reaction at 40 °C, the absorbance was measured at 680 nm using a Multiskan SkyHigh microplate reader (Thermo Fisher Scientific, Waltham, MA, USA). A standard curve was constructed using L-tyrosine, and one unit of protease activity (U/g) was defined as the amount of enzyme required to catalyze the production of 1 μg of L-tyrosine per minute.

Lipase activity was determined using the G0902F Lipase Activity Assay Kit (Suzhou Greys Biotechnology Co., Ltd., Suzhou, China) according to the manufacturer’s instructions. One unit (U/g) of lipase activity was defined as the amount of enzyme required to catalyze the production of 1 nmol of *p*-nitrophenol per minute.

### 2.5. Determination of Physicochemical Properties

The pH value of each sample was measured directly using a Testo 205 portable pH meter (Testo Inc., Lenzkirch, Germany), which was calibrated with standard buffer solutions (pH 4.01 and 7.01) before use. Water activity was measured using an SFY-30 water activity meter (Shanghai Guanya Precision Instruments Co., Ltd., Shanghai, China) after equilibrating 5 g of sample at 25 °C for 8 min. For the water-holding capacity (WHC) analysis, the sample (G_1_) was wrapped in three layers of filter paper, placed in a 50 mL centrifuge tube, and centrifuged at 4 °C and 5000× *g* for 20 min. The sample was then quickly removed and weighed to obtain mass G_2_, and WHC (%) was calculated as WHC (%) = (G_2_/G_1_) × 100. Color parameters including lightness (L*), redness (a*), and yellowness (b*) were measured using an NH350 portable colorimeter (Shenzhen Sanenshi Technology Co., Ltd., Shenzhen, China).

### 2.6. Free Amino Acids

Free amino acids were determined according to the method of Xi et al., with minor modifications [18]. Each 2 g sample was homogenized with ultrapure water and allowed to stand for 24 h. The resulting supernatant was then mixed with an equal volume of 5% (*w*/*v*) sulfosalicylic acid solution to precipitate proteins. After centrifugation at 6000× *g* for 10 min, the supernatant was collected and concentrated using a rotary evaporator. The residue was dissolved in 1 mL of sodium citrate buffer and filtered through a 0.45 μm membrane filter. The filtrate was analyzed using a Biochrom30+ amino acid analyzer (Biochrom Ltd., Cambridge, UK) equipped with a post-column ninhydrin detector. Quantification was performed using the external standard method with a mixed standard solution of 16 amino acids (Sigma-Aldrich, St. Louis, MO, USA). Information on the purity and CAS numbers of the amino acid standards is summarized in Supplementary Table S1.

### 2.7. Free Fatty Acids

Free fatty acid levels were determined according to the method of Lin et al., with slight modifications [19]. Each 100 mg sample was mixed with 4 mL of chloroform, vortexed for 30 s, and centrifuged at 3500× *g* for 15 min at room temperature. The lower organic phase was collected, and the extraction was repeated twice using 2 mL of dichloromethane each time. The combined organic extracts were dried under a gentle stream of nitrogen with mild heating (~35 °C). Derivatization was then carried out by adding 2 mL of methylation reagent, vortexing for 30 s, and incubating in a water bath at 80 °C for 2 h. The container was loosely covered with gauze to minimize gas loss. After cooling to room temperature, 2 mL of n-hexane and 1 mL of water were added and thoroughly vortexed. The mixture was centrifuged at 2000× *g* for 5 min, and the upper organic phase was collected and evaporated under a gentle stream of nitrogen with mild heating. An appropriate volume of iso-octane was added based on the sample concentration, vortexed, and left to stand. The final solution was filtered through a 0.22 μm PTFE syringe filter before being transferred to a sample vial for analysis. Quantification was performed using the external standard method with a mixed standard solution containing 36 fatty acids (Sigma-Aldrich, St. Louis, MO, USA).

A chromatographic analysis was performed using an Agilent 6890 gas chromatograph (Agilent Technologies, Santa Clara, CA, USA) equipped with a CP-Sil 88 capillary column (100 m × 0.25 mm × 0.25 μm). A 1 μL sample was injected with a split ratio of 10:1, and helium was used as the carrier gas at a constant flow rate of 1.0 mL/min. The initial oven temperature was set at 100 °C and held for 5 min, then increased at a rate of 4 °C/min to 240 °C, and held at 240 °C for 15 min. Mass spectrometric analysis was conducted using an Agilent 5977 mass spectrometer (Agilent Technologies, Santa Clara, CA, USA) equipped with an electron ionization (EI) source and operated using the MassHunter Workstation software package. The analysis was conducted in selected ion monitoring (SIM) mode, with the ion source temperature set at 260 °C, quadrupole temperature at 150 °C, ionization voltage at 70 eV, and scan rate at 57.7 cycles per second. In SIM mode, only specific characteristic ions (*m*/*z*) for each target fatty acid were monitored, all of which fall within the scan window of *m*/*z* 30 to 550. Information on the purity, CAS numbers, monitored *m*/*z* values, and retention times of the fatty acid standards is summarized in Supplementary Table S2. Compound identification was performed by matching retention times and mass spectral data with those of authentic standards. Data pre-processing—including baseline correction, peak detection, alignment, and retention time calibration—was performed using the Quant-My-Way Agilent B.09.00 software program.

### 2.8. Volatile Compound

Volatile compounds were analyzed using the Pegasus GC HRT4D+ system (LECO Corporation, St. Joseph, MI, USA). Each 5 g sample was transferred into a 20 mL headspace autosampler vial. Solid-phase microextraction (SPME) was performed using a CTC 3-in-1 autosampler equipped with a 50/30 μm CAR/PDMS/DVB fiber (Supelco, Bellefonte, PA, USA). Extraction was performed at 60 °C for 30 min, followed by fiber conditioning at 250 °C for 3 min and analyte desorption at 250 °C for 2 min. Data acquisition was performed using the LECO Pegasus 4D workstation and analyzed with the integrated ChromaTOF^®^ 4.61.1.0 software program. Chromatographic peak widths were set to 24 s and 0.2 s for the first and second dimensions, respectively. Peaks with a signal-to-noise ratio (S/N) greater than 200 were automatically detected, integrated, and deconvoluted. The linear retention index (LRI) was calculated based on the retention times of a C_5_–C_30_ n-alkane series and matched against the NIST 2017 and Wiley 9 databases. Compounds with both similarity and reverse match scores ≥ 800 were selected for final identification.

### 2.9. Electronic Tongue Analysis

Electronic tongue analysis was conducted according to the method of Liu et al., with modifications [20]. Each 10 g sample was homogenized and heated in a water bath until the core temperature reached 40 °C. Subsequently, 80 mL of water at the same temperature was added and further mixed for 1 min. After centrifugation at 3000× *g* for 10 min at 4 °C, the supernatant was collected for electronic tongue testing. A SA402B taste sensing system (Insent, Kanagawa, Japan) was used to characterize the taste profile. The system was equipped with five lipid membrane sensors—SB2CA0 (sourness), SB2C00 (bitterness), SB2AE1 (astringency), SB2AAE (umami), and SB2CT0 (saltiness)—along with an Ag/AgCl reference electrode.

Before measurement, the taste sensors and reference electrode were activated for 24 h in a reference solution (30 mmol/L KCl + 0.3 mmol/L tartaric acid) and saturated KCl solution, respectively, to stabilize signal responses. Measurements were conducted at room temperature (25 ± 2 °C) following a standardized five-step procedure: cleaning, equilibration, sample measurement, re-cleaning, and aftertaste recording. Sensors were first cleaned with cation and anion cleaning solutions for 90 s, rinsed in two reference solutions for 120 s each, and the baseline potential (Vr) was recorded for 30 s. The sensors were then immersed in the sample solution to measure the sample potential (Vs, 30 s), followed by two brief rinses in reference solution (3 s), after which the aftertaste potential (Vr′, 30 s) was recorded. Each sample was tested in quadruplicate, with the first reading discarded and the mean of the remaining three used to ensure signal stability and reproducibility. Data were processed using the instrument’s dedicated software and expressed as relative taste intensity values. A principal component analysis (PCA) was applied to identify taste distribution patterns and visualize differences among sample groups.

### 2.10. Data Statistics and Analysis

All experiments were independently performed in triplicate to ensure reliability, and the results were expressed as mean ± standard deviation (mean ± SD). Statistical analyses were conducted using the SPSS 25.0 software package (IBM, Armonk, NY, USA), with significance set at *p* < 0.05. To validate the statistical assumptions, normality was tested using the Shapiro–Wilk test, and the homogeneity of variance was assessed using Levene’s test. If both conditions were met, a one-way analysis of variance (ANOVA) was performed, followed by Waller–Duncan’s multiple range test for post hoc comparisons. When the assumptions were not satisfied, the Kruskal–Wallis nonparametric test was applied, and pairwise comparisons were conducted based on mean ranks. A principal component analysis (PCA) was performed using the Anaconda software program (Anaconda, Inc., Austin, TX, USA). Graphs were generated using Origin 2021 (OriginLab Corporation, Northampton, MA, USA) and the Chiplot platform (https://www.chiplot.online/, accessed on 25 April 2025).

## 3. Results and Discussion

### 3.1. Effects of Different Carbon and Nitrogen Sources on D. hansenii Growth and Enzymatic Activities

**Figure 1:**
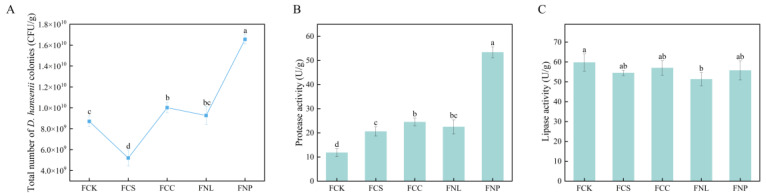
Total colony count of *D. hansenii* (**A**), protease activity (**B**), and lipase activity (**C**) in the sausage medium after 5 days of fermentation. FCK: control; FCS: sucrose; FCC: corn starch; FNL: leucine; FNP: soy protein isolates. Different letters indicate significant differences (*p* < 0.05).

The growth and metabolic characteristics of microorganisms are strongly influenced by the type and concentration of carbon and nitrogen sources in the medium, a factor closely related to the metabolic regulation mechanisms of the fermenting strain [21]. As shown in Figure 1A, the supplementation of different exogenous carbon and nitrogen sources significantly affected the proliferation of *D. hansenii*. Under carbon source treatment, the FCC group (corn starch) exhibited a colony count of 1.00 × 10^10^ CFU/g, which was significantly higher than that of the control group, 8.70 × 10^9^ CFU/g (*p* < 0.05). This result was likely due to the slow-release property of corn starch, which may provide a stable energy supply to support sustained cell activity. In contrast, the FCS group (sucrose) showed a 40.23% decrease in colony count, which may be associated with osmotic stress. According to Clara Navarrete et al., under high-salt stress conditions, *D. hansenii* tends to allocate energy to maintaining cellular homeostasis rather than promoting rapid cell division [22]. Under nitrogen source treatment, both the FNL group (leucine) and the FNP group (soy protein isolate) significantly increased colony counts. The FNP group exhibited the highest colony count at 1.66 × 10^10^ CFU/g, representing a 90.23% increase compared to the control (*p* < 0.05). This enhanced proliferation may be attributed to the higher nitrogen content (~15%) and more complex nitrogenous composition of soy protein isolate, compared to leucine (~10.7%). Upon hydrolysis, soy protein isolate releases a broad spectrum of amino acids and peptides, which may more effectively support the synthesis of nucleic acids, proteins, and cofactors essential for cell proliferation. In contrast, the FNL group was supplemented with a single amino acid. Although leucine modestly enhanced growth, its limited nitrogen profile may not have met the broader metabolic requirements of the strain.

Protease and lipase are two crucial enzymes produced by *D. hansenii* during fermentation; they are involved in the hydrolysis of proteins and fats, respectively, thereby enhancing the flavor profile of fermented meat products [23]. As shown in Figure 1B, protease activity was significantly higher in all treatment groups compared to the control group, with the FNP group exhibiting the highest activity at 55.38 U/g, approximately 5-fold higher than the control and 2-fold higher than the other treatment groups (*p* < 0.05). These results indicate that exogenous carbon and nitrogen supplementation enhanced the protease activity in *D. hansenii*, with various sources potentially affecting the strain through distinct nutritional pathways or physiological effects. Carbon sources can indirectly stimulate protease synthesis by providing carbon skeletons and an efficient energy supply to sustain high metabolic activity, while nitrogen sources can boost metabolic activity and enhance protease expression by supplying essential nitrogen [24]. In addition, soy protein isolate, as a high-molecular-weight protein, may serve as a substrate for extracellular proteases, potentially enhancing protease activity through substrate–enzyme recognition [25]. The elevated activity observed in the treatment groups may facilitate the release of amino acids and peptides—key precursors of flavor compounds—enhancing the umami and aromatic complexity of the final product.

Figure 1C shows that exogenous carbon and nitrogen sources did not significantly affect the lipase activity of *D. hansenii*. This may be because lipase is an inducible enzyme, and its synthesis typically depends on the presence of specific lipid substrates acting as inducers [26]. It is possible that the carbon and nitrogen sources used in this experiment lacked the specific components required to induce lipase expression. However, since the lipid profile of the carbon and nitrogen sources was not measured in this experiment, this inference remains to be validated. The *D. hansenii* strain used in this experiment exhibited inherently high baseline lipase activity, and all treatment groups had lipase activity exceeding 50 U/g.

### 3.2. Physicochemical Properties

#### 3.2.1. pH, Water Activity, and Water-Holding Capacity

**Figure 2:**
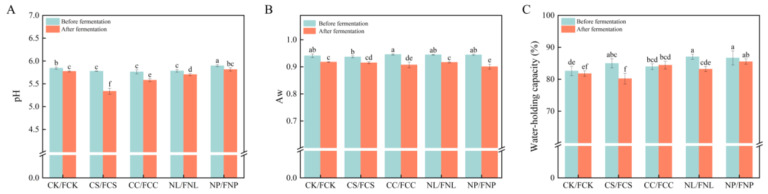
pH (**A**), Aw (**B**), and water-holding capacity (**C**) of the sausage medium before and after fermentation. CK: control; CS: sucrose; CC: corn starch; NL: leucine; NP: soy protein isolates. FCK, FCS, FCC, FNL, and FNP represent the samples after fermentation. Different letters indicate significant differences between groups (*p* < 0.05).

pH, water activity, and water-holding capacity are important indicators for evaluating the flavor and quality of fermented meat products. They also reflect microbial regulation of the substrate’s microenvironment through metabolism. As shown in Figure 2A, the pH value of all treatment groups significantly decreased after fermentation (*p* < 0.05). Among the groups, the carbon source treatments showed a more pronounced decrease in pH, with the FCS group reaching the lowest pH of 5.33. This may be due to the rapid metabolism of sucrose by *D. hansenii*, producing acidic byproducts like lactic and acetic acids [27]. In contrast, the pH decrease in FCC group was slower, which can be attributed to the gradual degradation characteristics of corn starch, a complex polysaccharide. The pH in the nitrogen source treatment groups remained relatively stable, especially in the FNP group, where the pH was maintained between 5.90 and 5.81. This stability may be due to the buffering capacity of amino acids such as aspartic acid and glutamic acid, released during the microbial metabolism of soy protein isolates. These amino acids help neutralize some of the acidic metabolic byproducts, thereby maintaining a relatively stable pH [28].

Figure 2B,C illustrate changes in water activity and water-holding capacity of the sausage matrix before and after fermentation. Both the FCC group (complex carbon source) and the FNP group (complex nitrogen source) significantly reduced water activity and increased water-holding capacity (*p* < 0.05). Among these, corn starch exhibited a strong water absorption capacity, continuously adsorbing free water and forming a stable protein–water network structure [29]. Soy protein isolates, possessing both water absorption and gel-forming properties, released degradation products, such as small peptides and hydrophilic amino acids, during microbial metabolism. These products further enhance water binding and improve the structural stability of the system [30]. In contrast, the simple carbon and nitrogen source treatment groups (FCS and FNL) showed no significant differences in water activity or water-holding capacity compared to the control group. This may be because neither the carbon nor the nitrogen sources were able to form stable protein–water networks, nor did they provide the spatial structure and hydrophilic groups required for effective water retention [31].

#### 3.2.2. Color

**Figure 3:**
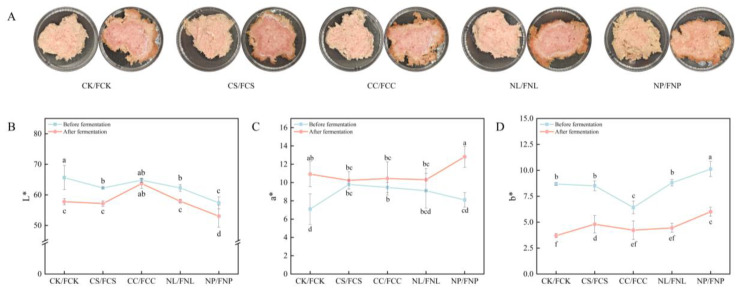
Appearance changes in the sausage medium before and after fermentation (**A**), and color changes: L* (**B**), a* (**C**), b* (**D**). CK: control; CS: sucrose; CC: corn starch; NL: leucine; NP: soy protein isolates. FCK, FCS, FCC, FNL, and FNP represent the samples after fermentation. Different letters indicate significant differences (*p* < 0.05).

Color is a key sensory indicator of the quality of fermented meat products and directly influences consumer acceptance. Figure 3A shows the appearance of the sausage matrix before and after fermentation, while Figure 3B, Figure 3C, and Figure 3D illustrate the specific changes in L*, a*, and b* values, respectively.

As shown in Figure 3B, fermentation led to a decrease in L* in the control group, while the addition of corn starch in the FCC group effectively slowed this decline. Corn starch, with its water absorption and expansion properties, reduced transparency and formed a uniform structure with proteins, enhancing light scattering and reflection, thereby maintaining a higher L* value [32]. The soy protein isolate in the FNP group, with its inherent yellowish color, may undergo browning during fermentation, leading to lower L* values before and after fermentation. Figure 3C shows that the a* value in the FNP group was significantly higher than in the other groups (*p* < 0.05). The relatively stable pH observed in the FNP group may help explain the preserved color. Previous studies have suggested that increased acidity may accelerate myoglobin oxidation and cause discoloration [33]. Figure 3D illustrates that fermentation caused a decrease in b* values for all groups. During fermentation, the increased NaCl concentration decreased oxygen solubility in the system, promoting the formation of high-iron myoglobin, which led to a decrease in b* [34]. The yellow color inherent in soy protein isolates resulted in higher b* values in the FNP group both before and after fermentation.

In summary, supplementation with exogenous carbon and nitrogen sources had varying effects on the growth characteristics, enzyme activities, and physicochemical properties of *D. hansenii* in the fermented sausage model. Among the different treatments, the complex carbon source FCC group (corn starch) and the complex nitrogen source FNP group (soy protein isolates) exhibited the most significant improvements. Both groups significantly increased the colony count and protease activity of *D. hansenii*, while maintaining high lipase activity. Additionally, improvements in the color attributes and water-holding capacity of the fermented sausage were likely due to their macromolecular structures. In contrast, the simple carbon source FCS group (sucrose) and the simple nitrogen source FNL group (leucine) showed fewer improvements. The subsequent section of this manuscript focuses on the effects of corn starch and soy protein isolates on the accumulation of amino acids, fatty acids, and volatile flavor compounds during *D. hansenii* fermentation.

### 3.3. Amino Acid Analysis

Amino acids are important flavor precursors in fermented meat products, participating in enzymatic reactions and microbial metabolic processes. They are also essential nutrients for the human body and significantly contribute to improving the nutritional value of the product [35]. Table 1 shows the effect of different carbon and nitrogen sources on the amino acid profile. The total free amino acid (FAA) content in the FCC group was 258.37 mg/100 g, with no significant difference compared to the control group. This suggests that although corn starch increased protease activity, the hydrolysis products did not accumulate significantly in the substrate, which aligns with the findings of Ding et al. [14]. *D. hansenii* may have rapidly utilized the hydrolyzed amino acids for cell proliferation or anabolic metabolism, leading to limited accumulation in the medium. Moreover, previous studies suggest that glucose can induce carbon catabolite repression, potentially affecting microbial amino acid metabolism. Since corn starch is degraded into glucose, it is possible that this mechanism also played a role in the current fermentation system.

The total free amino acid (FAA) content in the FNP group was 391.63 mg/100 g, corresponding to a 53.28% increase compared to the control group. This increase is primarily due to the effective degradation of soy protein isolates by *D. hansenii* during fermentation, which provides a rich source of amino acids and short peptides, promoting amino acid accumulation. Additionally, as a complex nitrogen source, soy protein isolates avoided triggering nitrogen catabolite repression (NCR), which stimulated the strain to secrete more proteases, enhancing protein degradation and its conversion [36]. Furthermore, when nitrogen sources are limited, the inactivation of the TOR (target of rapamycin) signaling pathway may promote the degradation and utilization of non-preferred nitrogen sources, further enhancing the synthesis and accumulation of FAAs [37]. Therefore, the significant increase in free amino acid levels in the FNP group was likely due to the combined effects of raw material degradation, the strain’s high baseline protease activity, and nitrogen metabolism regulation.

Amino acids not only serve as important flavor precursors but also undergo chemical reactions to contribute to the formation of complex flavors. In the FNP group, umami FAAs and sweet FAAs increased by 66.67% and 65.26%, respectively, likely contributing to the enhancement of umami and sweetness in the sausage. Furthermore, in the FNP group, the levels of all essential amino acids, except for methionine (which is naturally deficient in soy protein isolates), were significantly increased (*p* < 0.05). Essential amino acids improve the nutritional profile of food by providing the body with necessary amino acids, promoting protein synthesis, boosting immune function, and supporting nervous system health [38,39]. Therefore, they play a key role in improving the health benefits of food. Overall, the higher free amino acid levels in the FNP group suggest a stronger flavor potential and greater nutritional value.

### 3.4. Fatty Acid Analysis

The extent of fat hydrolysis and the composition of fatty acids are key factors influencing both the flavor and nutritional quality of fermented meat products. In fermented meat matrices, *D. hansenii* secretes lipase to catalyze fat hydrolysis, producing free fatty acids (FFAs), which are important flavor precursors [2]. In preliminary tests, the strain used in this study also exhibited notable lipolytic activity. Based on these findings, this section explores the effects of exogenous carbon and nitrogen sources on the fatty acid profile in a sausage model.

The free fatty acid content in each group after 5 days of fermentation is presented in Table 2. The total FFA content in the FCC group (1251.13 mg/100 g) and the FNP group (1342.92 mg/100 g) was significantly lower than that in the control group (1772.35 mg/100 g), corresponding to decreases of 29.4% and 24.2%, respectively (*p* < 0.05). Despite the reduction in total FFAs, the carbon and nitrogen source treatments retained sufficient levels of flavor precursors (>1000 mg/100 g). The fatty acid composition in all three groups was similar, with major components including oleic acid (cis) (30%), palmitic acid (25–26%), linoleic acid (16–17%), and stearic acid (13–14%), all of which are important precursors for aldehyde formation, such as nonanal and hexanal. Since there was no significant difference in lipase activity among the three groups, it suggests that the accumulation of free fatty acids may be influenced by factors such as raw material composition, lipid metabolism mechanisms, and the strain’s energy supply strategy.

The low free fatty acid levels in the FCC group may be attributable to the energy-providing function of corn starch. The additional energy supply reduced the strain’s reliance on fat as an energy source, thereby lowering the fat hydrolysis rate. Moreover, glucose, a product of corn starch degradation, may activate the CCR mechanism in *D. hansenii*, thereby inhibiting fat catabolism [40]. The reduced level of free fatty acids in the FNP group may also result from the energy-yielding function of soy protein isolate, which could reduce the strain’s dependence on lipid catabolism. In addition, serine—a degradation product of soy protein—has been identified as a key precursor for phospholipid synthesis [41]. Based on the observation of the highest colony count in the FNP group, it is speculated that lipid-derived metabolites may have been preferentially directed toward membrane construction and biomass production, rather than accumulating in the medium.

The saturated fatty acid (SFA) content in the FCC and FNP groups decreased by 29.86% and 25.58%, respectively, compared to the control. The corresponding UFA/SFA ratios increased to 1.29 and 1.32, respectively. Given that a high SFA intake has been linked to increased cardiovascular risk, this compositional change may be of potential nutritional interest [42]. High levels of free fatty acids (FFAs) are generally associated with increased risks of lipid oxidation and rancidity [43]. The elevated FFA content observed in the control group may therefore have implications for product shelf life and flavor stability, although this would require further verification through TBARS and peroxide value measurements. Overall, the addition of exogenous carbon and nitrogen sources (corn starch and soy protein isolate) modulated the fatty acid profile of *D. hansenii*, potentially improving lipid composition while preserving flavor precursor availability, and indicating a favorable nutritional profile.

### 3.5. Volatile Compound Analysis

**Figure 4:**
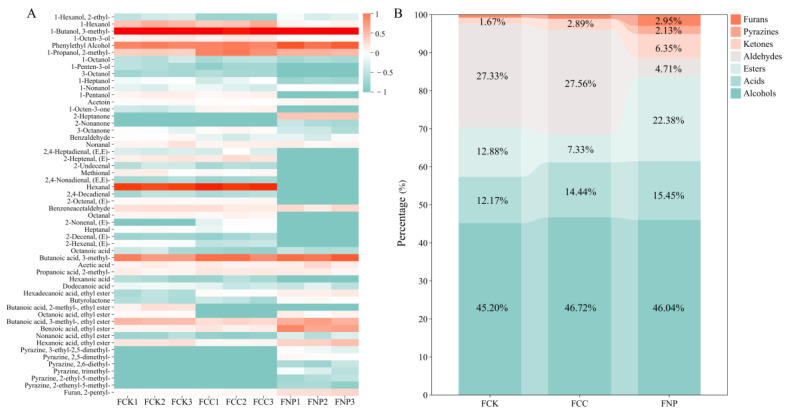
Abundance heatmap of volatile compounds (**A**) and stacked bar chart of relative content of each category of volatile compounds (**B**). The heatmap is color-coded from red to green, with red indicating relatively high content and green indicating relatively low content.

Volatile compounds play a crucial role in determining the flavor characteristics and consumer acceptance of fermented sausages. In this study, SPME-GC-MS was employed to analyze the differences in volatile compounds among the groups, and heatmaps of their relative contents, along with stacked bar charts for each compound category, were generated (Figure 4A,B). A total of 54 volatile compounds were detected, including 12 alcohols, 16 aldehydes, 6 acids, 5 ketones, 8 esters, 6 pyrazines, and 1 furan compound. Specifically, 45 compounds were detected in the control group, 44 in the FCC group, and 38 in the FNP group. Significant differences in the composition of volatile flavor compounds were observed among the groups (*p* < 0.05), suggesting that the addition of carbon and nitrogen sources significantly affected the accumulation of volatile compounds in *D. hansenii*-fermented sausages.

The relative contents of alcohols and acids were similar among the three groups, with alcohols being the most abundant volatile compounds in each group. These compounds accumulated in large quantities during the early stages of fermentation and served as precursors for key flavor compounds, such as esters and aldehydes [44]. Alcohols and acids primarily originated from yeast lipid oxidation, amino acid metabolism, and carbohydrate metabolism, with typically high thresholds, contributing to alcoholic, grassy, acidic, and cheesy aromas in fermented meat products [45]. The differences in alcohol content among the treatment groups may be attributed to the metabolic regulation characteristics of *D. hansenii*. In the FCC group, due to the lack of exogenous nitrogen sources, the strain may have relied more on sugar metabolism for energy, resulting in the production of alcohols, such as isobutanol [46]. In contrast, the high levels of FAAs in the FNP group may have triggered the Ehrlich pathway, which could convert amino acids into higher alcohols [47]. For instance, phenylethanol, a metabolic product of phenylalanine, was significantly increased in the FNP group (*p* < 0.05), contributing to rose and honey-like aroma characteristics.

Relatively high levels of aldehydes were observed in the FCC group (27.56%) and control group (27.33%). Aldehydes generally result from the oxidation and degradation of unsaturated fatty acids [1]. The high free fatty acid content in the control group served as a sufficient substrate for aldehyde accumulation. Although the free fatty acid content in the FCC group was lower, aldehyde intermediates might still have been generated through metabolic pathways such as sugar degradation [48]. In contrast, the aldehyde content in the FNP group was significantly lower (4.71%) than that in the other two groups (*p* < 0.05). The observed effect may be associated with the antioxidant activity of soy protein isolate hydrolysates. According to previous studies, these hydrolysates can contribute to lipid stabilization through mechanisms such as metal ion chelation, hydrogen donation, and free radical scavenging [49]. It is worth noting that aldehydes have a low sensory threshold, and even low concentrations can impart a fresh, herbal aroma to fermented meat products, whereas higher concentrations may result in greasy or rancid odors [50].

Esters are key flavor components in fermented meat products, characterized by low sensory thresholds and playing a crucial role in enhancing aroma. They are typically formed through the esterification of alcohols and acids, and yeasts can also catalyze the formation of ethyl esters via the alcohol acetyltransferase pathway, utilizing acetyl-CoA and higher alcohols [51]. In the FCC group, the relative content of esters decreased to 7.33%, which may be related to the CCR effect triggered by glucose, degraded from corn starch. This effect is suggested to further inhibit metabolic pathways in *D. hansenii* associated with the synthesis of higher alcohols and the expression of alcohol acetyltransferase, thereby limiting the generation and conversion of ester precursors and reducing ester accumulation, as reported in previous studies [7]. In contrast, the FNP group, by alleviating the NCR effect, may have enhanced the activity of metabolic pathways related to ester synthesis, leading to a significant increase in ester content, which reached 22.38%. The FNP group showed the substantial accumulation of volatile compounds such as ethyl hexanoate and ethyl benzoate, which are commonly associated with “fruity” and “floral” aromas in previous studies.

The relative content of ketones in the FCC and FNP groups increased to 2.89% and 6.35%, respectively. Ketones, which have a low sensory threshold, contribute to fruity, floral, and creamy aromas. They primarily originate from β-keto acid decarboxylation, incomplete β-oxidation of free fatty acids, and amino acid degradation. Some studies have found that carbon sources are converted into pyruvate through glycolysis, which can then be transformed into acetyl-CoA, and under specific metabolic conditions, short-chain ketones may be produced via an atypical pathway [52]. In the FNP group, the abundant nitrogen source may have maintained metabolic balance in *D. hansenii*, reducing its dependence on fat for energy. This increase in incomplete β-oxidation flux could have promoted the accumulation of medium-chain ketones, such as 2-heptanone, which have contributed to fruity aromas [53].

The FNP group also exhibited a significant accumulation of 2-pentylfuran (2.95%), a low-threshold compound known to contribute to plant-like aromas such as beany and green notes. Additionally, pyrazine compounds (2.13%) were detected exclusively in the FNP group. Pyrazines require carbohydrates as carbon source precursors and amino acids as nitrogen source precursors [54]. The elevated FAA levels in the FNP group may have provided sufficient substrates for non-enzymatic pyrazine synthesis, promoting the early accumulation of pyrazines during fermentation. Pyrazines have an extremely low flavor threshold and can contribute unique roasted and nutty aromas at low concentrations.

In summary, the FCC group generated aldehydes and ketones under conditions of low free fatty acid levels, contributing fresh, grassy flavor characteristics to the sausages. The relative content of esters, ketones, and pyrazines increased in the FNP group, with reduced aldehyde contents, leading to a complex and rich flavor profile with fruity, nutty, and other distinctive characteristics.

### 3.6. Taste Characteristics of Sausage Model

**Figure 5:**
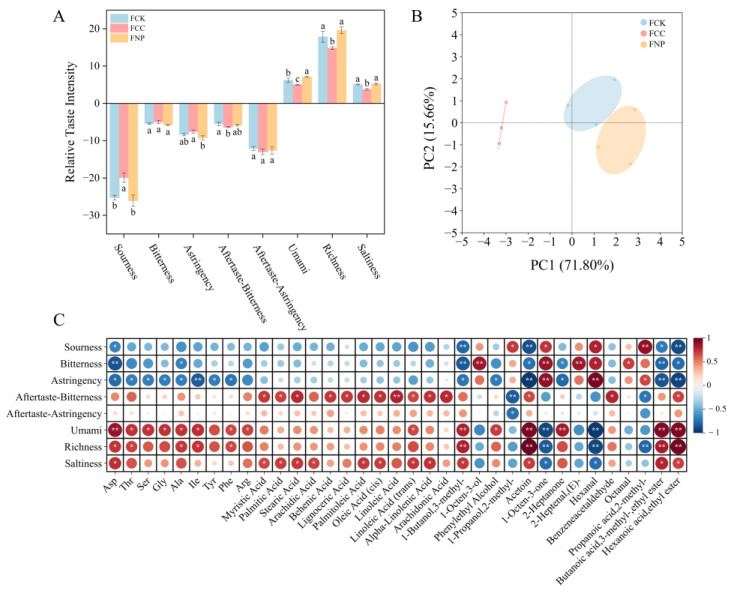
Taste characteristics of fermented sausage model, including bar chart of the electronic tongue (**A**), PCA plot of the electronic tongue (**B**), and correlation heatmap with major amino acids, fatty acids, and volatile compounds (**C**). Red indicates a positive correlation, blue indicates a negative correlation, and white indicates no correlation. Different letters under the same parameter indicate significant differences (*p* < 0.05); asterisks indicate significance levels: * *p* < 0.05, ** *p* < 0.01.

Electronic tongue technology was used to further examine the impact of corn starch and soy protein isolates on the flavor characteristics of a fermented sausage model. The electronic tongue simulates the human tongue’s ability to perceive key taste attributes, such as sourness, sweetness, saltiness, bitterness, and umami, offering an objective reflection of taste differences between samples. As shown in Figure 5A, the FCC group (corn starch) exhibited significantly increased sourness and decreased saltiness, which can be attributed to the accumulation of organic acids produced by an active glycolysis pathway and the lower degree of fat hydrolysis. The FNP group (soy protein isolates) exhibited a prominent umami response, attributable to its high content of glutamic acid and flavor peptides, which synergistically interact with ester compounds to enhance the overall flavor. As shown in Figure 5B, a principal component analysis (PCA) explained 87.46% of the total variance, effectively distinguishing the taste distribution characteristics among the different treatment groups. PC1 (71.80%) primarily differentiates between carbon source and nitrogen source treatments, while PC2 (15.66%) reflects differences in fermentation levels. The FNP and FCC groups were positioned on either side of the control group in the PCA space, suggesting that carbon and nitrogen sources may induce distinct taste clustering patterns through different metabolic regulation mechanisms. When combined with analyses of FAAs, free fatty acids, and volatile flavors, it can be inferred that the enhanced sourness in the FCC group was related to the acidic byproducts from active sugar metabolism, while the decreased saltiness was likely due to lower fat hydrolysis and limited salt-soluble protein degradation. An electronic tongue analysis indicated enhanced umami and taste richness in the FNP group, which may be associated with the accumulation of short peptides and FAAs, as well as abundant esters, pyrazines, and furans.

To further explore the relationship between flavor compounds and sensory taste attributes, a Spearman correlation analysis was performed to construct a correlation heatmap between 44 major volatile and non-volatile flavor compounds and 8 electronic tongue sensory characteristics (Figure 5C). The results showed that compounds such as isoamyl alcohol, 3-hydroxy-2-butanone, isobutyric acid, and ethyl isoamyl ester were positively correlated with umami, richness, and saltiness, suggesting their role in enhancing these taste attributes. Phenylethanol, 2-heptanone, and the umami amino acid glutamic acid also significantly contributed to the umami characteristic. Additionally, while free fatty acids enhance saltiness, they may also impart bitterness and a bitter aftertaste. Compounds such as 1-octen-3-one, hexanal, and octanal were positively correlated with astringency, bitterness, and sourness, indicating that these compounds could contribute to unpleasant sensory experiences at high concentrations.

In summary, the analysis of both the electronic tongue data and flavor compounds revealed a significant association between key metabolites and taste attributes. The FCC group exhibited a fresh, acidic taste with reduced saltiness, while the FNP group exhibited prominent umami and richness.

## 4. Conclusions

This study initially assessed the effects of simple and complex carbon and nitrogen sources on the fermentation characteristics of local *D. hansenii*. The results demonstrated that the complex carbon and nitrogen sources, including corn starch and soy protein isolates, were particularly effective. Both sources exhibited good water retention properties. Corn starch, serving as a slow-release carbon source, maintained a stable energy supply, boosting colony counts, protease activity, and product brightness. Soy protein isolates, providing a stable nitrogen source and alleviating the NCR effect, led to enhanced growth and protease secretion in *D. hansenii*. A further analysis revealed that corn starch accumulated aldehydes and ketones, contributing fresh flavor characteristics. Soy protein isolates, through raw material degradation, the synergistic action of *D. hansenii*’s high protease activity, and nitrogen metabolism regulation, increased the total free amino acid content, particularly enhancing umami and sweetness components. It also enriched esters, ketones, and pyrazines, while reducing aldehydes, resulting in a more intense and complex aroma profile. Notably, both carbon and nitrogen sources optimized the fatty acid profile, contributing to the improved nutritional value of the final product. In summary, supplementation with different exogenous carbon and nitrogen sources can regulate the fermentation characteristics of *D. hansenii* through distinct metabolic pathways, producing differentiated effects in promoting growth and enzyme activity, improving physicochemical properties, and optimizing flavor compound accumulation. The rational selection of carbon and nitrogen sources provides technical support for improving the quality and flavor of fermented sausages, as well as for achieving controlled flavor regulation in fermented meat products. In future studies, the fermentation characteristics of the strain can be further optimized by combining carbon and nitrogen sources and adjusting the carbon-to-nitrogen ratio.

## Figures and Tables

**Table 1:** Amino acid composition and content of the fermented sausage medium with different carbon and nitrogen sources.

Free Amino Acids	FCK	FCC	FNP
	mg/100 g		
Asp	3.30 ± 0.70 ^b^	2.40 ± 0.61 ^b^	6.47 ± 0.40 ^a^
Glu	20.85 ± 1.34 ^b^	22.63 ± 2.20 ^b^	34.03 ± 1.72 ^a^
Umami FAAs	24.30 ± 2.26 ^b^	25.03 ± 2.07 ^b^	40.50 ± 1.42 ^a^
Thr *	7.93 ± 0.67 ^b^	7.50 ± 0.75 ^b^	12.53 ± 0.71 ^a^
Ser	17.20 ± 0.71 ^b^	16.43 ± 1.80 ^b^	33.53 ± 0.72 ^a^
Gly	6.75 ± 0.35 ^b^	7.03 ± 0.61 ^b^	11.47 ± 1.20 ^a^
Ala	16.43 ± 0.81 ^b^	15.03 ± 1.87 ^b^	25.77 ± 2.89 ^a^
Pro	9.50 ± 0.72 ^c^	12.13 ± 0.76 ^b^	14.70 ± 1.04 ^a^
Sweet FAAs	59.30 ± 1.32 ^b^	58.13 ± 1.52 ^b^	98.00 ± 0.66 ^a^
Val *	19.30 ± 0.85 ^b^	20.33 ± 1.44 ^b^	30.23 ± 1.06 ^a^
Met *	8.37 ± 0.91 ^a^	8.80 ± 1.00 ^a^	8.37 ± 0.50 ^a^
Ile *	14.83 ± 0.31 ^b^	14.60 ± 1.47 ^b^	23.17 ± 1.21 ^a^
Leu *	19.80 ± 1.25 ^b^	20.37 ± 0.71 ^b^	37.17 ± 1.29 ^a^
Tyr	15.80 ± 0.50 ^b^	16.33 ± 0.99 ^b^	25.37 ± 2.83 ^a^
Phe *	24.03 ± 1.34 ^b^	25.23 ± 1.92 ^b^	34.73 ± 1.36 ^a^
His	6.77 ± 0.49 ^a^	7.33 ± 0.85 ^a^	9.67 ± 2.31 ^a^
Lys *	25.20 ± 2.56 ^b^	27.03 ± 1.42 ^b^	40.53 ± 1.04 ^a^
Arg	10.87 ± 1.86 ^b^	10.07 ± 1.53 ^b^	17.43 ± 0.61 ^a^
Bitter FAAs	145.57 ± 1.12 ^b^	150.10 ± 3.72 ^b^	226.67 ± 9.18 ^a^
Cys	25.07 ± 0.06 ^a^	25.10 ± 0.56 ^a^	26.47 ± 1.10 ^a^
Total FAAs	255.50 ± 3.91 ^b^	258.37 ± 20.59 ^b^	391.63 ± 10.72 ^a^

“*” indicates essential amino acids. Different letters under the same parameter indicate significant differences (*p* < 0.05).

**Table 2:** Fatty acid composition and content of the fermented sausage medium with different carbon and nitrogen sources.

Free Fatty Acids	FCK	FCC	FNP
	mg/100 g		
Butyric Acid	0.21 ± 0.08 ^a^	0.08 ± 0.01 ^b^	0.07 ± 0.01 ^b^
Caproic acid	0.04 ± 0.02 ^a^	0.02 ± 0.02 ^ab^	0.01 ± 0.01 ^b^
Caprylic Acid	0.17 ± 0.02 ^a^	0.10 ± 0.06 ^a^	0.11 ± 0.01 ^a^
Capric Acid	2.39 ± 0.23 ^a^	1.73 ± 0.79 ^a^	1.87 ± 0.09 ^a^
Undecylic acid	0.08 ± 0.01 ^a^	0.06 ± 0.03 ^a^	0.07 ± 0.01 ^a^
Lauric Acid	3.17 ± 0.28 ^a^	2.28 ± 1.03 ^a^	2.47 ± 0.11 ^a^
Tridecylic acid	0.07 ± 0.01 ^a^	0.05 ± 0.02 ^a^	0.05 ± 0.01 ^a^
Myristic Acid	42.36 ± 3.33 ^a^	29.69 ± 1.28 ^b^	32.11 ± 1.03 ^b^
Pentadecanoic Acid	1.19 ± 0.09 ^a^	0.80 ± 0.35 ^a^	0.85 ± 0.02 ^a^
Palmitic Acid	459.87 ± 16.92 ^a^	327.85 ± 7.89 ^b^	345.56 ± 10.99 ^b^
Heptadecanoic Acid	6.06 ± 0.60 ^a^	4.01 ± 1.85 ^a^	4.39 ± 0.26 ^a^
Stearic Acid	256.87 ± 14.02 ^a^	175.67 ± 4.62 ^b^	187.31 ± 5.16 ^b^
Arachidic Acid	5.98 ± 0.52 ^a^	3.75 ± 0.36 ^b^	4.33 ± 0.21 ^b^
Heneicosanoic acid	0.13 ± 0.02 ^a^	0.07 ± 0.03 ^b^	0.09 ± 0.01 ^b^
Behenic Acid	0.39 ± 0.04 ^a^	0.27 ± 0.07 ^b^	0.32 ± 0.01 ^ab^
Tricosanoic acid	0.08 ± 0.01 ^a^	0.05 ± 0.01 ^b^	0.06 ± 0.01 ^b^
Lignoceric Acid	0.22 ± 0.04 ^a^	0.14 ± 0.02 ^a^	0.19 ± 0.09 ^a^
Myristoleic Acid	0.50 ± 0.03 ^a^	0.35 ± 0.20 ^a^	0.35 ± 0.05 ^a^
Palmitoleic Acid	61.95 ± 4.86 ^a^	44.27 ± 2.91 ^b^	48.74 ± 2.17 ^b^
Margaroleic Acid	4.97 ± 1.96 ^a^	3.22 ± 1.64 ^a^	3.26 ± 0.09 ^a^
Oleic Acid (trans)	3.88 ± 0.31 ^a^	2.59 ± 1.23 ^a^	2.85 ± 0.12 ^a^
Oleic Acid (cis)	533.37 ± 22.61 ^a^	376.65 ± 6.25 ^b^	403.13 ± 13.20 ^b^
Gadoleic Acid	25.23 ± 2.33 ^a^	16.18 ± 1.27 ^b^	17.63 ± 0.95 ^b^
Erucic Acid	1.52 ± 0.29 ^a^	1.37 ± 0.14 ^a^	1.36 ± 0.02 ^a^
Nervonic Acid	0.41 ± 0.05 ^a^	0.34 ± 0.14 ^ab^	0.23 ± 0.03 ^b^
Linoleic Acid	299.71 ± 7.21 ^a^	216.81 ± 7.01 ^c^	238.00 ± 9.29 ^b^
Linoleic Acid (trans)	0.16 ± 0.01 ^a^	0.11 ± 0.02 ^b^	0.16 ± 0.01 ^a^
γ-Linolenic Acid	1.16 ± 0.10 ^a^	0.79 ± 0.37 ^a^	0.88 ± 0.07 ^a^
Alpha-Linolenic Acid	21.50 ± 2.19 ^a^	15.06 ± 0.94 ^b^	16.89 ± 0.92 ^b^
Eicosadienoic Acid	20.38 ± 1.35 ^a^	13.04 ± 2.45 ^b^	14.47 ± 0.61 ^b^
Dihomo-gamma-Linolenic Acid	2.69 ± 0.19 ^a^	1.82 ± 0.68 ^b^	2.09 ± 0.03 ^ab^
Erucic acid	1.52 ± 0.29 ^a^	1.37 ± 0.14 ^a^	1.36 ± 0.02 ^a^
Eicosatrienoic Acid	3.63 ± 0.38 ^a^	2.13 ± 0.85 ^b^	2.44 ± 0.28 ^b^
Arachidonic Acid	9.93 ± 0.21 ^a^	8.04 ± 1.27 ^b^	8.81 ± 0.15 ^ab^
Eicosapentaenoic Acid	0.26 ± 0.02 ^a^	0.18 ± 0.02 ^b^	0.19 ± 0.01 ^b^
Docosadienoic Acid	0.29 ± 0.04 ^a^	0.18 ± 0.08 ^b^	0.19 ± 0.01 ^ab^
SFAs	779.29 ± 34.73 ^a^	546.63 ± 15.60 ^b^	579.88 ± 17.63 ^b^
MUFAs	631.83 ± 29.89 ^a^	444.96 ± 10.67 ^b^	477.55 ± 16.33 ^b^
PUFAs	361.23 ± 10.92 ^a^	259.54 ± 12.69 ^c^	285.49 ± 10.64 ^b^
UFAs	993.06 ± 40.61 ^a^	704.50 ± 23.07 ^b^	763.04 ± 26.89 ^b^
UFAs/SFAs	1.27 ± 0.01 ^b^	1.29 ± 0.02 ^ab^	1.32 ± 0.01 ^a^
Total FFAs	1772.35 ± 75.34 ^a^	1251.13 ± 36.90 ^b^	1342.92 ± 44.36 ^b^

SFAs: saturated fatty acids; MUFAs: monounsaturated fatty acids; PUFAs: polyunsaturated fatty acids; UFAs: unsaturated fatty acids. Different letters under the same parameter indicate significant differences (*p* < 0.05).

## Data Availability

The original contributions presented in the study are included in the article, and further inquiries can be directed to the corresponding author.

## Supplementary Materials

The following supporting information can be downloaded at: https://www.mdpi.com/article/10.3390/foods14111840/s1, Table S1. Free Amino Acid Standard Information; Table S2. Free Fatty Acid Standard Information; Table S3. Volatile compound composition and content of fermented sausage culture medium with different carbon and nitrogen sources; Figure S1. Representative chromatograms of free fatty acids in fermented samples from different treatment groups: A, Standard sample; B, control (FCK); C, corn starch (FCC); D, soy protein isolate (FNP).
