# Supplementary material for: Soy Protein Isolate Supplementation Favorably Regulates the Fermentation Characteristics of Debaryomyces hansenii and Flavor Profile in a Sausage Model
Source: Foods. 2025 May 22;14(11):1840. doi: 10.3390/foods14111840 (PMC12154309; doi:10.3390/foods14111840)
Supplement: Supplementary file 1 [file foods-14-01840-s001.zip › Supplementary image.pdf]

Figure Captions

Figure S1. Representative chromatograms of free fatty acids in fermented samples from different treatment groups: A, Standard sample; B, control (FCK); C, corn starch (FCC); D, soy protein isolate (FNP).

Figure S1

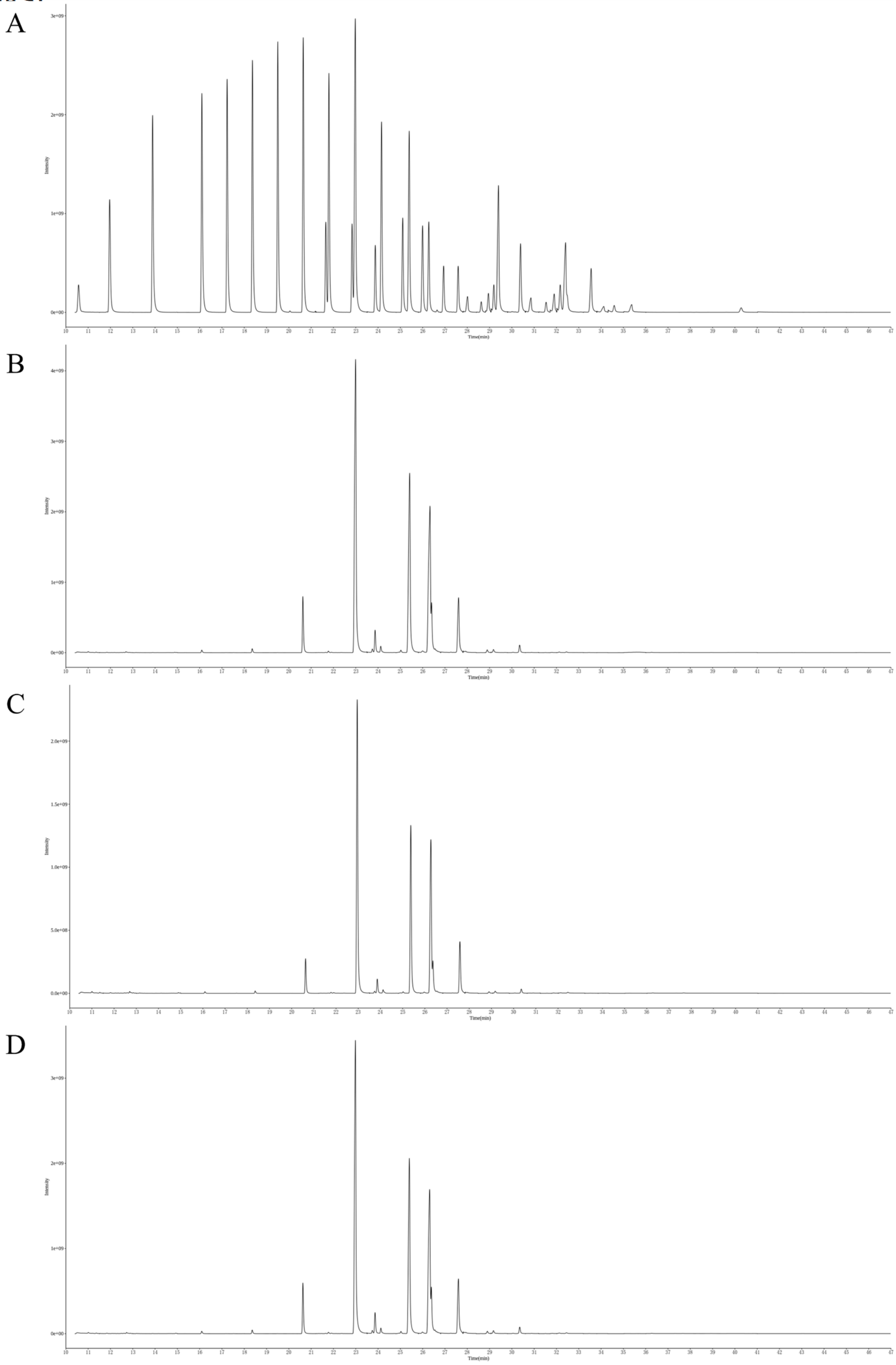

A
B
C
D
Intensity
Time(min)
3e+09
2e+09
1e+09
0e+00
4e+09
2.0e+09
1.5e+09
1.0e+09
5.0e+08
0.0e+00
