# Supplementary material for: Soy Protein Isolate Supplementation Favorably Regulates the Fermentation Characteristics of Debaryomyces hansenii and Flavor Profile in a Sausage Model
Source: Foods. 2025 May 22;14(11):1840. doi: 10.3390/foods14111840 (PMC12154309; doi:10.3390/foods14111840)
Supplement: Supplementary file 1 [file foods-14-01840-s001.zip › Supplementary table.pdf]

Table Captions

Table S1. Free Amino Acid Standard Information

Table S2. Free Fatty Acid Standard Information

Table S3. Volatile compound composition and content of fermented sausage culture medium with different carbon and nitrogen sources

Table S1

Free Amino Name	Abbreviation	CAS Number	Molecular Formula
Aspartic acid	Asp	56-84-8	$C_4H_7NO_4$
Glutamic acid	Glu	56-86-0	$C_5H_9NO_4$
Threonine	Thr	72-19-5	$C_4H_9NO_3$
Serine	Ser	56-45-1	$C_3H_7NO_3$
Glycine	Gly	56-40-6	$C_2H_5NO_2$
Alanine	Ala	56-41-7	$C_3H_7NO_2$
Proline	Pro	147-85-3	$C_5H_9NO_2$
Valine	Val	72-18-4	$C_5H_{11}NO_2$
Methionine	Met	63-68-3	$C_5H_{11}NO_2S$
Isoleucine	Ile	73-32-5	$C_6H_{13}NO_2$
Leucine	Leu	61-90-5	$C_6H_{13}NO_2$
Tyrosine	Tyr	60-18-4	$C_9H_{11}NO_3$
Phenylalanine	Phe	63-91-2	$C_9H_{11}NO_2$
Histidine	His	71-00-1	$C_6H_9N_3O_2$
Lysine	Lys	56-87-1	$C_6H_{14}N_2O_2$
Arginine	Arg	74-79-3	$C_6H_{14}N_4O_2$
Cysteine	Cys	52-90-4	$C_3H_7NO_2S$

All standard compounds were obtained from Sigma-Aldrich (purity $\geqslant$ 99%)

Table S2

Fatty Acid Name	Abbreviation	CAS Number	Molecular Formula	Rt/min	Quantified ion (m/z)
Butyric acid	C4:0	107-92-6	$C_4H_8O_2$	10.521	74.0
Hexanoic acid	C6:0	142-62-1	$C_6H_{12}O_2$	11.951	74.0
Octanoic acid	C8:0	124-07-2	$C_8H_{16}O_2$	13.880	74.0
Decanoic acid	C10:0	334-48-5	$C_{10}H_{20}O_2$	16.090	74.0
Undecanoic acid	C11:0	112-37-8	$C_{11}H_{22}O_2$	17.224	74.0
Lauric acid	C12:0	143-07-7	$C_{12}H_{24}O_2$	18.358	74.0
Tridecanoic acid	C13:0	638-53-9	$C_{13}H_{26}O_2$	19.491	74.0
Myristic acid	C14:0	544-63-8	$C_{14}H_{28}O_2$	20.630	74.0
Myristoleic acid	C14:1	544-64-9	$C_{14}H_{26}O_2$	21.646	74.0
Pentadecanoic acid	C15:0	1002-84-2	$C_{15}H_{30}O_2$	21.777	74.0
cis-10-Pentadecenoic acid	C15:1	84743-29-3	$C_{15}H_{28}O_2$	22.823	96.0
Palmitic acid	C16:0	57-10-3	$C_{16}H_{32}O_2$	22.938	74.0
Palmitoleic acid	C16:1	373-49-9	$C_{16}H_{30}O_2$	23.857	96.0
Heptadecanoic acid	C17:0	506-12-7	$C_{17}H_{34}O_2$	24.126	87.0
cis-10-heptadecenoic acid	C17:1	29743-97-3	$C_{17}H_{32}O_2$	25.083	69.0
Stearic acid	C18:0	57-11-4	$C_{18}H_{36}O_2$	25.351	74.0
Elaidic acid	C18:1n9t	112-79-8	$C_{18}H_{34}O_2$	25.958	96.0
Oleic acid	C18:1n9c	112-80-1	$C_{18}H_{34}O_2$	26.239	96.0
Linolelaidic acid	C18:2n6t	506-21-8	$C_{18}H_{32}O_2$	26.912	96.0
Linoleic acid	C18:2n6c	60-33-3	$C_{18}H_{32}O_2$	27.568	96.0
Arachidic acid	C20:0	506-30-9	$C_{20}H_{40}O_2$	27.940	55.0
Gamma-Linolenic acid	C18:3n6	506-26-3	$C_{18}H_{30}O_2$	28.609	87.0
11-Eicosenoic acid	C20:1	5561-99-9	$C_{20}H_{38}O_2$	28.898	67.0
Linolenic acid	C18:3n3	463-40-1	$C_{18}H_{30}O_2$	29.170	55.0
Henicosanoic acid	C21:0	2363-71-5	$C_{21}H_{42}O_2$	29.315	74.0
11C,14C-Eicosadienoic acid	C20:2	2091-39-6	$C_{20}H_{36}O_2$	30.349	95.0
Docosanoic acid	C22:0	112-85-6	$C_{22}H_{44}O_2$	30.751	55.0
cis-8,11,14-Eicosatrienoic acid	C20:3n6	1783-84-2	$C_{20}H_{34}O_2$	31.501	87.0
Erucic acid	C22:1n9	112-86-7	$C_{22}H_{42}O_2$	31.816	67.0

cis-11,14,17-Eicosatrienoic acid	C20:3n3	17046-59-2	$C_{20}H_{34}O_2$	32.124	55.0
Tricosanoic acid	C23:0	2433-96-7	$C_{23}H_{46}O_2$	32.291	87.0
Arachidonic acid	C20:4n6	506-32-1	$C_{20}H_{32}O_2$	32.444	55.0
13C,16C-Docosadienoic acid	C22:2	17735-98-7	$C_{22}H_{40}O_2$	33.487	95.0
Lignoceric acid	C24:0	557-59-5	$C_{24}H_{48}O_2$	33.980	81.0
Eicosapentaenoic acid	C20:5n3	10417-94-4	$C_{20}H_{30}O_2$	32.559	74.0
Nervonic acid	C24:1	506-37-6	$C_{24}H_{46}O_2$	35.258	67.0
cis-4,7,10,13,16,19-Docosahexaenoic acid	C22:6n3	6217-54-5	$C_{22}H_{32}O_2$	40.254	55.0

All standard compounds were obtained from Sigma-Aldrich (purity ≥ 99%)

Table S3

Volatile compounds	CAS	Retention Index	FCK	FCC	FNP
			Relative Content (%)		
1-Hexanol, 2-ethyl-	104-76-7	1481.3	0.29±0.05[b]	0.11±0.01[c]	0.39±0.06[a]
1-Hexanol	111-27-3	1345.9	5.46±0.71[a]	5.12±1.26[a]	1.25±0.22[b]
1-Butanol, 3-methyl-	123-51-3	1202.4	21.41±1.74[ab]	18.41±1.31[b]	25.33±3.08[a]
1-Octen-3-ol	3391-86-4	1442.1	1.67±0.45[a]	1.90±0.45[a]	0.61±0.07[b]
Phenylethyl Alcohol	60-12-8	1912.6	9.36±0.32[b]	9.36±0.46[b]	11.61±1.10[a]
1-Propanol, 2-methyl-	78-83-1	1097.6	3.96±0.27[c]	9.21±0.83[a]	5.88±0.41[b]
1-Octanol	111-87-5	1549.6	0.25±0.06[a]	0.21±0.04[a]	0.16±0.02[a]
1-Penten-3-ol	616-25-1	1159	0.20±0.04[a]	0.11±0.05[b]	ND
3-Octanol	589-98-0	1385.2	0.14±0.03[a]	0.17±0.03[a]	ND
1-Heptanol	111-70-6	1447.3	0.48±0.03[a]	0.41±0.10[a]	0.12±0.02[b]
1-Nonanol	143-08-8	1651.9	0.40±0.05[b]	0.37±0.06[b]	0.69±0.07[a]
1-Pentanol	71-41-0	1245.1	1.58±0.27[a]	1.31±0.28[a]	ND
Acetoin	513-86-0	1284	0.86±0.14[b]	0.63±0.05[b]	1.32±0.24[a]
1-Octen-3-one	4312-99-6	1298.4	0.31±0.03[b]	1.31±0.13[a]	ND
2-Heptanone	110-43-0	1182.1	ND	ND	4.57±0.05
2-Nonanone	821-55-6	1385.5	ND	ND	0.2±0.07
3-Octanone	106-68-3	1252.6	0.51±0.13[b]	0.96±0.19[a]	0.26±0.07[b]
Benzaldehyde	100-52-7	1528.5	0.71±0.24[a]	0.38±0.05[b]	0.4±0.07[b]
Nonanal	124-19-6	1390.7	1.81±0.74[a]	1.44±0.27[a]	1.43±0.45[a]
2,4-Heptadienal, (E,E)-	4313-03-5	1466.1	0.34±0.02[a]	0.41±0.12[a]	ND
2-Heptenal, (E)-	18829-55-5	1323.2	2.04±0.46[a]	2.51±0.65[a]	ND
2-Undecenal	2463-77-6	1752.5	0.27±0.06[a]	0.18±0.03[b]	ND
Methional	3268-49-3	1456.7	1.37±0.66[a]	0.91±0.24[a]	ND
2,4-Nonadienal, (E,E)-	5910-87-2	1704.5	0.17±0.04[a]	0.13±0.03[a]	ND
Hexanal	66-25-1	1080	13.91±0.56[b]	15.46±0.88[a]	ND
2,4-Decadienal	2363-88-4	1813.6	0.21±0.06[a]	0.2±0.03[a]	ND
2-Octenal, (E)-	2548-87-0	1429.1	0.93±0.08[b]	1.08±0.11[a]	ND
Benzeneacetaldehyde	122-78-1	1648.1	2.76±0.10[a]	2.23±0.46[a]	2.88±0.70[a]
Octanal	124-13-0	1286.5	1.37±0.07[a]	1.2±0.22[a]	ND
2-Nonenal, (E)-	18829-56-6	1535.6	ND	0.51±0.14	ND
Heptanal	111-71-7	1182.4	0.72±0.18[a]	0.51±0.10[a]	ND
2-Decenal, (E)-	3913-81-3	1644.5	0.12±0.01[a]	0.14±0.06[a]	ND
2-Hexenal, (E)-	6728-26-3	1219	0.62±0.15[a]	0.26±0.02[b]	ND
Octanoic acid	124-07-2	2063.4	0.26±0.08[a]	0.11±0.03[b]	0.22±0.05[ab]
Butanoic acid, 3-methyl-	503-74-2	1668	8.33±1.12[b]	10.08±1.12[ab]	11.02±1.01[a]

Acetic acid	64-19-7	1465.7	1.52 ± 0.26^{a}	1.68 ± 0.28^{a}	2.3 ± 0.90^{a}
Propanoic acid, 2-methyl-	79-31-2	1569.4	1.48 ± 0.36^{a}	2.1 ± 0.18^{a}	1.63 ± 0.47^{a}
Hexanoic acid	142-62-1	1849.7	0.16 ± 0.06^{a}	0.15 ± 0.06^{a}	ND
Dodecanoic acid	143-07-7	2483.4	0.43 ± 0.01^{a}	0.32 ± 0.06^{ab}	0.29 ± 0.08^{b}
Hexadecanoic acid, ethyl ester	628-97-7	2246.3	0.20 ± 0.05^{c}	0.75 ± 0.08^{b}	1.79 ± 0.31^{a}
Butyrolactone	96-48-0	1637.9	0.20 ± 0.04^{b}	0.23 ± 0.07^{b}	0.81 ± 0.18^{a}
Butanoic acid, 2-methyl-, ethyl ester	7452-79-1	1052.9	2.32 ± 0.71	ND	ND
Octanoic acid, ethyl ester	106-32-1	1428.9	0.84 ± 0.05^{a}	ND	1.28 ± 0.56^{a}
Butanoic acid, 3-methyl-, ethyl ester	108-64-5	1068.6	5.38 ± 0.24^{b}	3.25 ± 0.35^{c}	6.41 ± 0.79^{a}
Benzoic acid, ethyl ester	93-89-0	1669.8	1.31 ± 0.13^{b}	1.87 ± 0.41^{b}	7.66 ± 1.22^{a}
Nonanoic acid, ethyl ester	123-29-5	1530	0.15 ± 0.06^{b}	0.07 ± 0.01^{b}	0.31 ± 0.09^{a}
Hexanoic acid, ethyl ester	123-66-0	1228.1	2.47 ± 0.24^{b}	1.15 ± 0.11^{c}	4.13 ± 0.82^{a}
Pyrazine, 3-ethyl-2,5-dimethyl-	13360-65-1	1440.7	ND	ND	0.46 ± 0.09
Pyrazine, 2,5-dimethyl-	123-32-0	1318.7	ND	ND	0.82 ± 0.16
Pyrazine, 2,6-diethyl-	13067-27-1	1430.3	ND	ND	0.18 ± 0.09
Pyrazine, trimethyl-	14667-55-1	1399.5	ND	ND	0.35 ± 0.12
Pyrazine, 2-ethyl-5-methyl-	13360-64-0	1387.4	ND	ND	0.19 ± 0.06
Pyrazine, 2-ethenyl-5-methyl-	13925-08-1	1494.8	ND	ND	0.13 ± 0.06
Furan, 2-pentyl-	3777-69-3	1229.7	0.75 ± 0.03^{b}	1.06 ± 0.12^{b}	2.95 ± 0.37^{a}

The relative contents were calculated based on peak area normalization. ND: Not Detected. Different letters under the same parameter indicate significant differences ($p < 0.05$).
